# Parental Attitudes and Hesitancy About COVID-19 vs. Routine Childhood Vaccinations: A National Survey

**DOI:** 10.3389/fpubh.2021.752323

**Published:** 2021-10-13

**Authors:** Mohamad-Hani Temsah, Abdullah N. Alhuzaimi, Fadi Aljamaan, Feras Bahkali, Ayman Al-Eyadhy, Abdulkarim Alrabiaah, Ali Alhaboob, Fahad A. Bashiri, Ahmad Alshaer, Omar Temsah, Rolan Bassrawi, Fatimah Alshahrani, Yazan Chaiah, Ali Alaraj, Rasha Assad Assiri, Amr Jamal, Mohammed A. Batais, Basema Saddik, Rabih Halwani, Fahad Alzamil, Ziad A. Memish, Mazin Barry, Sarah Al-Subaie, Jaffar A. Al-Tawfiq, Khalid Alhasan

**Affiliations:** ^1^Pediatric Department, College of Medicine, King Saud University, Riyadh, Saudi Arabia; ^2^Division of Pediatric Cardiology, Department of Cardiac Sciences, College of Medicine, King Saud University, Riyadh, Saudi Arabia; ^3^Division of Pediatric Cardiology, Department of Cardiac Sciences, King Saud University Medical City, Riyadh, Saudi Arabia; ^4^Critical Care Department, College of Medicine, King Saud University, Riyadh, Saudi Arabia; ^5^College of Medicine, Alfaisal University, Riyadh, Saudi Arabia; ^6^Division of Infectious Diseases, Department of Internal Medicine, College of Medicine, King Saud University and King Saud University Medical City, Riyadh, Saudi Arabia; ^7^Department of Medicine, College of Medicine, Qassim University, Qassim, Saudi Arabia; ^8^Dr Sulaiman Al Habib Medical Group, Riyadh, Saudi Arabia; ^9^Department of Basic Medical Sciences, College of Medicine, Princess Nourah bint Abdulrahman University, Riyadh, Saudi Arabia; ^10^Department of Family and Community Medicine, King Saud University Medical City, Riyadh, Saudi Arabia; ^11^Evidence-Based Health Care & Knowledge Translation Research Chair, King Saud University, Riyadh, Saudi Arabia; ^12^Sharjah Institute of Medical Research, University of Sharjah, Sharjah, United Arab Emirates; ^13^Department of Community and Family Medicine, College of Medicine, University of Sharjah, Sharjah, United Arab Emirates; ^14^Department of Clinical Sciences, College of Medicine, University of Sharjah, Sharjah, United Arab Emirates; ^15^Research and Innovation Center, King Saud Medical City, Ministry of Health, Riyadh, Saudi Arabia; ^16^College of Medicine, Alfaisal University, Riyadh, Saudi Arabia; ^17^Hubert Department of Global Health, Rollins School of Public Health, Emory University, Atlanta, GA, United States; ^18^Specialty Internal Medicine and Quality Department, Johns Hopkins Aramco Healthcare, Dhahran, Saudi Arabia; ^19^Infectious Disease Division, Department of Medicine, Indiana University School of Medicine, Indianapolis, IN, United States; ^20^Infectious Disease Division, Department of Medicine, Johns Hopkins University School of Medicine, Baltimore, MD, United States

**Keywords:** vaccine hesitancy scale, COVID-19 Vaccine, childhood vaccines, national survey data, parental vaccine acceptance, parental vaccination intention, parental vaccine concerns

## Abstract

**Objectives:** To quantify parental acceptance of the COVID-19 vaccine and assess the vaccine hesitancy (VH) for COVID-19 vs. childhood vaccines.

**Methods:** Eight vaccine hesitancy scale (VHS) items, adopted from WHO's Strategic Advisory Group of Immunization (SAGE), were used to assess VH for COVID-19 vaccine vs. routine childhood vaccines. We distributed the online survey to parents with the commence of the national childhood COVID-19 vaccination program in Saudi Arabia.

**Results:** Among 3,167 parents, 47.6% are decided to vaccinate their children against COVID-19. The most common reasons for refusal were inadequate safety information (69%) and worry about side effects (60.6%). Parents have a significantly greater positive attitudes toward children's routine vaccines vs. the COVID-19 vaccine, with higher mean VHS (±SD) = 2.98 ± 0.58 vs. 2.63 ± 0.73, respectively (*p*-value < 0.001). Parents agreed more that routine childhood vaccines are more essential and effective as compared to the COVID-19 vaccine (Cohen's D: 0.946, and 0.826, consecutively; *T*-test *p*-value < 0.00). There is more parental anxiety about serious side effects of the COVID-19 vaccine vs. routine childhood vaccines (Cohen's *D* = 0.706, *p*-value < 0.001). Parents who relied on the Ministry of Health information were more predicted (OR = 1.28, *p*-value = 0.035) to intend to vaccinate as opposed to those who used the WHO website (OR = 0.47, −53%, *p*-value < 0.001). In a multivariate logistic regression analysis, the factors associated with intention to vaccinate children were parents who received COVID-19 vaccine, older parents, having children aged 12–18, and parents with lower education levels.

**Conclusions:** Significant proportion of parents are hesitant about the COVID-19 vaccine because they are less confident in its effectiveness, safety, and whether it is essential for their children. Relying on the national official healthcare authority's website for the source of information was associated with increased acceptance of childhood COVID-19 vaccination. As parental intention to vaccinate children against COVID-19 is suboptimal, healthcare authorities could boost vaccine uptake by campaigns targeting hesitant parents.

## Introduction

Coronavirus Disease 2019 (Covid-19) was declared a global pandemic on March 11, 2020 (1, 2). It was quickly recognized that vaccine development and deployment were among the most promising intervention strategies to mitigate the spread of Covid-19 (3). By July 25th, 2021, the total number of Covid-19 cases had reached more than 193 million worldwide, with 4.2 million deaths (4). Children's infections represent around 14% of the Covid-19 cases (5). While most SARS-CoV-2 infections in children are mild compared to those in adults (6), multiple cases of multisystem inflammatory syndrome in children and adolescents were reported (7). As herd immunity needed to halt the pandemic is estimated to be 75%, global healthcare authorities implemented various strategies that are best tailored to their society needs and availability of vaccines to maximize vaccine delivery for all ages (8). Though several Covid-19 vaccines were developed and deployed during the past year, healthcare authorities cannot curb the oandemic without widespread vaccine uptake (9).

The World Health Organization (WHO) has previously listed vaccine hesitancy, described as the reluctance or refusal of vaccines, as one of the major threats to public health before the current Covid-19 pandemic (10). Several Covid-19 vaccine surveys showed variable vaccine acceptance in different settings, with many healthcare workers (HCWs) being unsure of or not planning to receive the vaccine (11–13). Vaccine hesitancy (VH) is defined as “a behavior, influenced by a number of factors including issues of confidence (do not trust a vaccine or a provider), complacency (do not perceive a need for a vaccine or do not value the vaccine), and convenience (access)” (14).

As of July 2021, the Kingdom of Saudi Arabia (KSA) has reported 517,000 cases and 8,000 deaths; more than 25 million have received at least one dose of the vaccine, including around one million aged 12–18, and accounting for more than 70% of the population (15). The Covid-19 vaccine for children aged 12–18 years old was launched in the country on June 25th, 2021 (16). When vaccine hesitancy creates anxiety among adults, they may refuse or worry about having their children vaccinated. For children under the age of 18 years, parents are usually the decision-makers regarding their children's vaccinations; hence, it is important to understand parents' intentions, attitudes about the Covid-19 vaccine, and related barriers and facilitators. There have been no studies investigating the effects of parental acceptance of the Covid-19 vaccination for their children in KSA.

## Methods

We employed a modified version of the WHO's Strategic Advisory Group of Experts on Immunization Vaccine Hesitancy Scale (VHS) (14) to improve the congruency and consistency of the scale to better fit the current Covid-19 situation. The tool was initially developed in 2015 and built upon extensive global pilot data of indicators for vaccine hesitancy; though a relatively recent instrument, the VHS has been used in various regions. It is utilized to assess parental vaccine hesitancy among childhood and adolescent vaccines, including the annual influenza vaccine.

The wording was slightly revised from the original VHS to tackle both the Covid-19 vaccine and childhood vaccination. In addition, all items except “New vaccines carry more risks than older vaccines” were asked twice: once concerning the COVID-10 vaccine and once in general, to create a point of comparison for our analysis.

We opted to omit the question “All childhood vaccines offered by the government program in my community are beneficial” to maximize the relevance of our questionnaire to better suit the study population. In a similar fashion to Kempe et al. (17), we altered the Likert scale into a 4-point scale, excluding the “neutral or not sure” option, as recent evidence suggests that a 4-point scale without the choice of neutrality reduces the potential for social conformity. For the possible causes of Covid-19 vaccine refusal and sources of information about vaccines, we adopted the previously published survey from KSA (18, 19).

The adapted survey was then translated and back-translated into the Arabic language. The final bilingual survey (Appendix 1) was piloted among 52 parents. Overall, the items were reliable and were understood equally reliably by people, with Cronbach's alpha = 0.87.

### Data Collection

We used the Snowball technique through various social media platforms as a modality of rapid subject recruitment and data sampling for the emergency health research during the rise of the second Covid-19 wave (20). The invitation link emphasized the inclusion criteria and voluntary participation, as well as the privacy of data in not seeking any individual identifiers.

### Statistical Analyses

The mean and standard deviation were used to describe continuous measured variables, and the frequency and percentages were used for describing the categorically measured variables. The multiple response dichotomies analysis was applied to the measured variables with more than one option (“tick all that applies” questions). The chi-squared (χ2)-test of independence was used to assess the correlations between categorically measured variables and the One-way ANOVA test was used to assess the statistical significance of mean differences on metric variables across the levels of categorical outcomes of more than two levels; however, a corrected chi-squared and One-way ANOVA tests were used when the statistical assumptions for these tests were found to be violated. The mean parental attitude (VHS) score toward vaccines was computed by averaging the eight items comprising the scale after reverse ordering the negatively-worded statements so that a higher score denoted a better attitude toward vaccines, then attitude VHS scores were dichotomized into low vs. high hesitance toward vaccines based on a cut off value (=3 points); thus, people with a mean attitude score lower than 3 VHS attitude points were considered to have high hesitance and people with a ≥ 3 VHS score were considered to have low hesitance toward vaccines. The paired samples *t*-test was used to compare people's attitudes toward routine and Covid-19 vaccines. Cohen's d cut limit for effect size was used (21).

The Multivariate Binary Regression analysis was used to assess the statistical significance of the predictors of people's odds of preferring their children's vaccination against the Covid-19 disease. The association between the parental odds of preferring the vaccine with their sociodemographic and other relevant predictor variables was expressed as an Odds Ratio with associated 95% confidence interval. The alpha significance level was considered at the 0.050 level. The SPSS IBM statistical analysis program Version#21 was used for the data analysis.

## Results

### Parental Sociodemographic Characteristics

The sociodemographic characteristics of the 3,167 parents residing in the KSA who were included in the analysis is shown in Table 1.

**Table 1:** Parental sociodemographic characteristics.

**Variable**	***N***	**%**
**Sex**		
Mother	2,059	65
Father	1,108	35
**Age group**		
18–34 years	504	15.9
35–44 years	1,482	46.8
45–54 years	855	27
55–64 years	285	9
= >65 years	41	1.3
**Marital state**		
Married	3,000	94.7
Single parent (widow, divorced)	167	5.3
**Educational Level**		
High school or less	351	11.1
College/University degree	2,463	77.8
Higher studies	353	11.1
**Households' monthly income**		
<5,000 SR (1,333 USD)	196	6.2
5,000–10,000 SR (1,333–2,666 USD)	515	16.3
More than 10,000 SR (2,666 USD)	1,825	57.6
Prefer not to answer	631	19.9
Number of Children, mean (SD)		4.10 (1.77)
Number of Children, Median		4
**Nationality**		
Saudi	2,471	78
Non-Saudi	696	22
**Employment**		
Unemployed	104	3.3
Retired	151	4.8
Housewife	301	9.5
Freelance/owns job	294	9.3
Employee	1,363	43
Healthcare Worker	954	30.1
**Do you have a child whose age is between 12 and 18 years?**		
No	1,069	33.8
Yes	2,098	66.2
**Was this child (aged 12–18 years) ever diagnosed with an organic or psychological illness?**		
No	3,028	95.6
Yes	139	4.4

*N = 3,167*.

*SR, Saudi Riyal; USD, US Dollar*.

**Figure 1:**
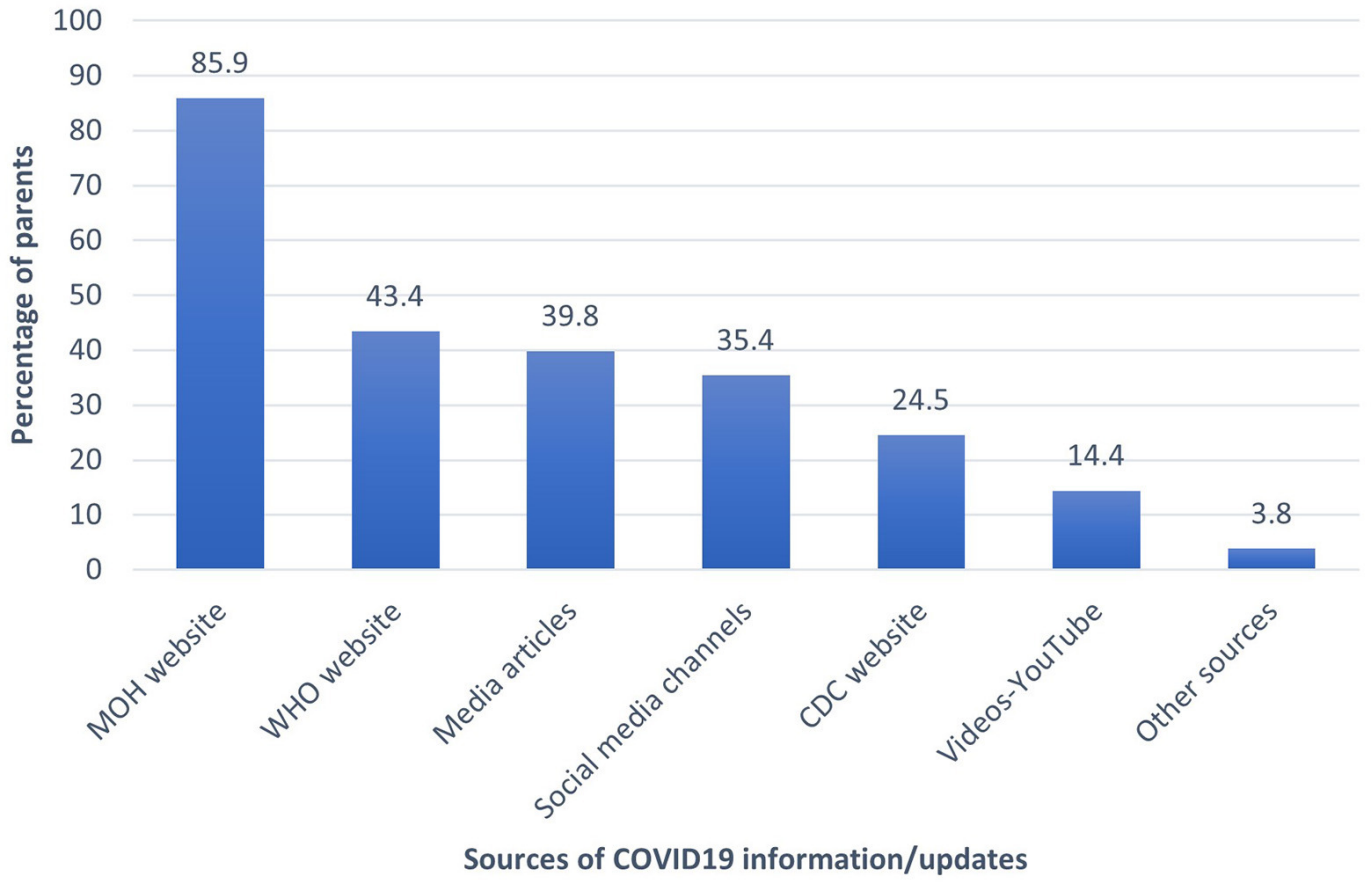
The parents' sources of COVID-19 information and updates.

Most parents (85.9%) used the Saudi Ministry of Health (MOH) website for Covid-19 information and vaccine updates, followed by the WHO online sources. The other parents' sources of information about Covid-19 are shown in Figure 1.

### Parental Intention to Vaccinate Their Children and Perceptions of Covid-19

86.7% of respondents had already received the Covid-19 vaccine themselves. Almost half of parents (47.6%) stated that they intend to vaccinate their children, while 32% were not, and another 20.5% stated they were undecided. The parents who indicated that they will not vaccinate their children against the Covid-19 were asked to select as many reasons as applicable for their decision. The top reasons were the inadequacy of information about the safety of the vaccines and their worry about potential side effects, 69 and 60.6%, respectively. Other reasons are listed in Table 2.

**Table 2:** Parental perceptions and behaviors related to COVID-19 and its vaccine.

**Survey question**	***N***	**%**
**As a parent, did you take the Covid-19 vaccine?**		
No	420	13.3
Yes	2,747	86.7
**Describe your family's commitment to the precautionary measures against the Covid-19 virus, mean (SD)**		4.26 (1.1)^*^
Rarely committed	124	3.9
Slightly committed	33	1
Medium commitment	766	24.2
Highly committed	206	6.5
Very Highly committed	2,038	64.4
**Are you willing/intending to give the COVID vaccine to your child (children)?**		
No	1,012	32
Unsure (undecided)	649	20.5
Yes	1,506	47.6
**Was anyone within your direct family infected with Covid-19?**		
No	890	28.1
Yes	2,277	71.9
**How severe were the symptoms of the infected person/s?** ***n*** **=** **802**		
Very mild/asymptomatic	76	9.5
Mild	361	45
Moderate	308	38.4
Severe	46	5.7
Very severe	2	0.2
Death	9	1.1
Parents' Generalized Anxiety GAD7 score, mean (SD)^**^		4.58 (4.80)
**What are your reasons for avoiding the Covid vaccine in children?** ***N*** **=** **992**		
Inadequate data about the safety of a new vaccine	684	69
I am against vaccines in general (or I avoid medications whenever possible)	212	21.4
Vaccine administration is painful or inconvenient	30	3
My child already had a COVID infection	104	10.5
A concern of adverse effects of the vaccine	601	60.6
A concern of acquiring Covid-19 from the vaccine	80	8.1
A concern of vaccine being ineffective from Covid mutations	161	16.2
Prior adverse reaction to the vaccine	63	6.4
I perceive my child as not at high risk to acquire Covid-19 infection	259	26.1
I perceive my child as not at high risk to develop complications if he/she get infected with Covid-19	264	26.6

*N = 3,167*.

* *Likert-like scale graded from 1 = rarely committed to 5 = very highly committed*.

** *GAD7 score: highest score 21*.

### Vaccine Hesitancy Scale for Covid-19 Vaccine vs. Childhood Vaccine

The parents' attitude toward the Covid-19 vaccine vs. childhood vaccine was measured using a modified VHS. The level of agreement was measured using 8 different statements for both the Covid-19 Vaccine and Childhood vaccine with a scale from 1 to 4 (Table 3).

**Table 3:** Paired sample *t*-test comparing parental attitudes toward routine and COVID-19 child vaccinations.

**Attitudes toward routine Covid-19 vaccine/childhood routine vaccines**	**Level of acceptance (1–4 scale) for childhood routine vaccine** mean (SD) ^*^	**Level of acceptance for Covid10 Vaccine (1–4 scale)** **mean (SD)**	Cohen's D^*^	***p*** -value^**^	Interpretation of difference^***^
Covid-19 vaccine/childhood routine vaccines is/are essential for my child's/children's health	3.37 (0.75)	2.60 (1.00)	0.946	<0.001	Large
Covid-19 vaccine/childhood routine vaccines is/are effective	3.26 (0.70)	2.64 (0.92)	0.826	<0.001	Large
Having my child vaccinated with Covid-19 vaccine/childhood routine vaccines is important for the health of others in my community	2.95 (0.89)	2.66 (0.98)	0.345	<0.001	Small
The information I acquire about Covid-19 vaccine/childhood routine vaccines from vaccine programs is credible	2.83 (0.77)	2.60 (0.86)	0.314	<0.001	Small
Getting Covid-19 vaccine/childhood routine vaccines is a good way to protect my child/children from disease	3.04 (0.81)	2.62 (0.93)	0.535	<0.001	Medium
Generally, I follow what my doctor or health care provider suggests about Covid-19 vaccine/childhood routine vaccines	3.14 (0.68)	2.79 (0.88)	0.486	<0.001	Small
I am anxious about serious adverse effects of Covid-19 vaccine/childhood routine vaccines	2.44 (0.84)	2.98 (0.87)	0.706	<0.001	High medium
My child/children do or don't need vaccines for diseases that are not common anymore	2.27 (0.84)	__	__	__	__
New vaccines (like Covid-19) bear more risks than older vaccines	__	2.61 (0.82)	__	__	__

* *Level of agreement 1–4: 1 strongly disagree, 2 disagree, 3 agree, 4 strongly agree*.

** *Paired samples t-test*.

*** *Cohen's d cut limit for effect size small 0.2, medium 0.5, large 0.8 and above*.

**Figure 2:**
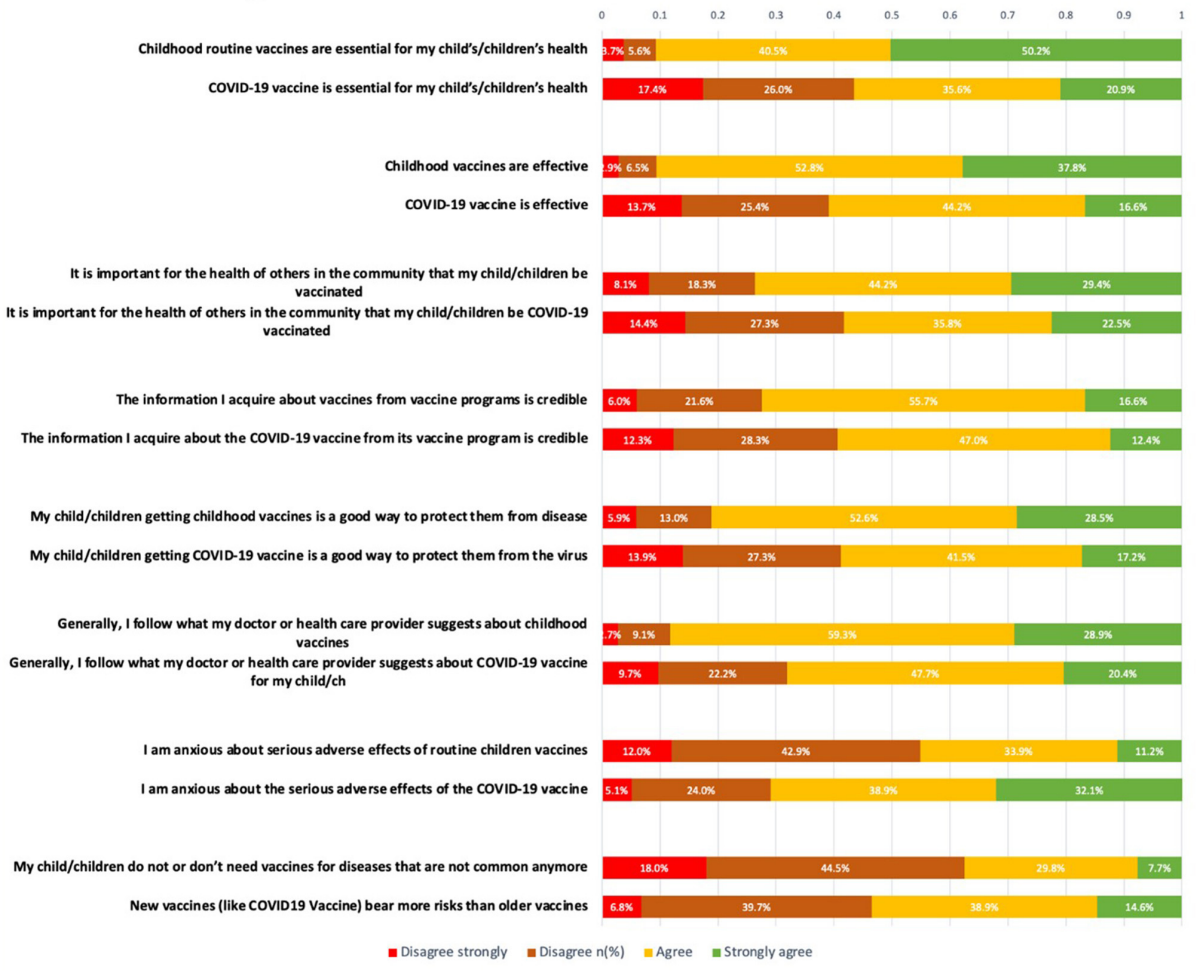
The parental attitudes toward childhood vaccines vs. COVID-19 vaccine.

The details for respondents' answers are shown in Figure 2. Parents agreed significantly more that routine childhood vaccines are essential and effective compared to the Covid-19 vaccine (Cohen's D: 0.946 and 0.826, consecutively, *P*-values < 0.001 using *T* test), while they were significantly anxious about the serious side effects of the Covid-19 vaccine vs. routine vaccines (significant medium difference Cohen's D = 0.706, *P*-value < 0.001). Furthermore, parents agree more that getting the childhood vaccines is important to protect their children compared to when the same statements were tested for the Covid-19 vaccine (Cohen's d: 0.535, *T*-test *p*-value < 0.001).

A Paired samples *t*-test was used to compare the parental mean attitudes toward routine and Covid-19 vaccines and showed that the parents had significantly greater positive attitudes toward the children's routine vaccines with a higher mean VHS score (SD) = 2.98 (0.58) compared to their attitudes toward the Covid-19 vaccines with VHS mean attitude (SD) = 2.63 (0.73) on average, denoting that they had a stronger agreement with the children's routine vaccines than the Covid-19 vaccines with a mean difference = 0.355 (95% C.I mean difference: 0.338–0.377), *p*-value < 0.001 (Appendix 1).

### Vaccine Hesitancy Scale vs. Parent Intention to Vaccinate Their Children

The parent intension to vaccinate or not vaccinate their children might be different than their underlying attitude.

The parental Covid-19 hesitancy (measured by VHS) converged inversely with the parents' intents to vaccinate their own children against the Covid-19 disease, *p* < 0.001 according to the chi-squared test of independence. About half of the sample (54.6%) had a VHS score ≥ 3 (not hesitant) for the Covid19 vaccine and 45% were hesitant toward the Covid-19 vaccine. On the other hand, 38.8 % were considered hesitant toward the routine vaccination.

Among those, 45% were hesitant about the Covid-19 vaccine, 50% did not intend to vaccinate their children, and another 27.5% were unsure; only 22.3% had decided to vaccinate their children. On the other hand, of those who were not hesitant according to the VHS score, 87.7% intended to vaccinate their children, 9.3% were unsure, and 3% refused the vaccine (Table 4).

**Table 4:** Bivariate Analysis of the Parental Willingness to COVID-19 Vaccinate their Child vs. Vaccine Hesitancy Scores (VHS).

	**Intention to vaccinate child against Covid-19**			**Total** **(*N*)**	**Test statistic**	***p*** **-value**
**Variable**	**No**	**Unsure**	**Yes**			
Attitude toward children's routine vaccines mean score	2.60 (0.611)	2.92 (0.45)	3.26 (0.44)		*F* (2,1604.8) = 482.87	<0.001
Attitude toward children's COVID vaccines mean score	1.92 (0.52)	2.48 (0.47)	3.17 (0.45)		*F* (2,1617.1) = 2,044.2	<0.001
**Covid-19 children's vaccine hesitancy**						
Not Hesitant: VHS score ≥3 points (*N*, %)	37 (3.0%)	114 (9.3%)	1,073 (87.7%)	1,732	χ2 (2) = 1,319.3	<0.001
Hesitant VHS score <3 (*N*, %)	975 (50.2%)	535 (27.5%)	433 (22.3%)	1,435		
**Routine children vaccine hesitancy**						
Not hesitant: VHS score ≥3 points (*N*, %)	281 (16.2%)	308 (17.8%)	1,143 (66.0%)	1,224	χ2 (2) = 583.04	<0.001
Hesitant VHS score <3 (*N*, %)	731 (50.9%)	341 (23.8%)	363 (25.3%)	1,943		

*N = 3,167*.

### Multivariate Analysis of Parents' Intention to Vaccinate Their Children With Covid-19 Vaccine

The predictors of parents' intention to vaccinate their children with the Covid-19 vaccine were analyzed using a multivariate binary logistic regression showing adjusted odds ratio for various parents' characteristics (Table 5). Parents who had already received the Covid-19 vaccine themselves were more likely to intend to vaccinate their children with an odds ratio of 3.62, *P*-value < 0.001. In addition, the households' number of children converged positively and significantly on their intentions to vaccinate them. Furthermore, if they were 12–18 years, parents were more decided (odds ratio 2.2, *P*-value < 0.001). The older the parent age group, the more likely they were to accept the Covid-19 vaccination for their children. The parents' gender, marital status, history of Covid-19 disease among family members, being unemployed (or student), and household income did not correlate significantly on their intention to vaccinate their children with the Covid-19 vaccine. The parents who had an educational level of high school or less were more likely to intend to vaccinate their children (odds ratio 2.09, *P* < 0.001).

**Table 5:** Multivariate binary logistic regression of parental intention to COVID-19 vaccinate their child.

**Variable**	**Multivariate adjusted odds ratio**	**95% C.I. for OR**		***p*** **-value**
		**Lower**	**Upper**	
Parent = Father	0.93	0.74	1.15	0.484
Parents Age group: 45–54 years	1.30	1.03	1.64	0.028
Parents Age group: 55–64 years	3.41	2.35	4.94	<0.001
Parents Age group: ≥65 years	3.68	1.36	9.98	0.011
Marital state = married/currently married or bound	0.76	0.50	1.15	0.195
Educational Level: High school or less	2.09	1.54	2.84	<0.001
Nationality = Saudi national	1.35	1.06	1.73	0.016
Job = Healthcare workers	1.26	1.00	1.59	0.046
Job = Unemployed/students	0.61	0.34	1.10	0.099
Household's monthly income group	1.06	0.97	1.15	0.176
Number of children	1.11	1.05	1.18	0.001
Has children aged 12–18 years	2.20	1.73	2.79	<0.001
History of Covid-19 disease among family members	1.05	0.84	1.30	0.683
Received the Covid-19 As a parent	3.62	2.58	5.09	<0.001
Source of information = National Ministry of Health (MOH) Website	1.28	1.02	1.60	0.035
Source of information = WHO website	0.47	0.38	0.59	<0.001
Hesitant about children's Covid-19 vaccines = yes	0.04	0.03	0.05	<0.001
Hesitant about routine child vaccines = yes	0.83	0.66	1.04	0.104
Constant	0.60			0.166

*Dependent Variable = Intends to vaccinate child against Covid-19 (No/Yes). Model overall statistical significance: χ2 (18) = 1,727.2, p-value < 0.001. Model Hosmer-Lemeshow G.O.F test χ2 (8) = 35.7, p = 0.737*.

People's nationalities converged significantly on their intentions to vaccinate their children against the Covid-19 disease; Saudi nationals compared to expatriates were found to be significantly more inclined to intend to vaccinate their children (OR = 1.35 times more, *P*-value = 0.016) on average. Also, people employed in health care jobs were found to be slightly more predicted (OR = 1.26 times, *p*-value = 0.046) to intend their own children's vaccination against Covid-19, compared to people in other jobs.

With regard to the relationship of source of information about Covid-19 and its vaccine, the multivariate analysis showed that the parents who relied on the Saudi MOH website information were found to be significantly more predicted (OR = 1.28 times more, *p*-value = 0.035) to intend the vaccination of their children. Conversely, people who relied on the WHO website updates were found to be significantly less predicted (53% times less predicted, *p*-value < 0.001) to intend to vaccinate their children against the Covid-19 disease. Unsurprisingly, the parental hesitancy toward the Covid-19 vaccine correlated significantly negatively with their intention to vaccinate their children against the Covid-19 disease. Parents who were hesitant about the Covid-19 vaccine were found to be significantly less predicted (96% times less predicted) to intend vaccinating their own children against the Covid-19 viral disease, compared those who were not hesitant *p*-value < 0.001, while their hesitancy toward the routine children's vaccinations did not converge significantly on their intentions to vaccinate their child against the Covid-19 disease.

## Discussion

As most countries strive to achieve Covid-19 herd immunity through widespread vaccination campaigns, KSA had achieved 70% vaccination rate for adults so far (16). According to the Saudi General Authority for Statistics, 11 million Saudis are younger than 19-years-old, representing 37% of the population; thus, vaccinating that age group is an essential step toward reaching that target (22). In late June of 2021, the Pfizer-BioNTech vaccine was approved for the 12–18 years age group in the KSA. Previous work by Alsubaie et al. has shown that 20% of parents in the KSA are hesitant to give routine immunization to their children (23). Interruption of routine immunization during the time of the COVID pandemic and the misleading information about the COVID vaccine had its impact on routine childhood vaccination programs, as observed in some studies (24, 25). VH has been found to be a major barrier to achieving herd immunity. A recent study showed that, depending on varying biological, environmental, and socio-behavioral factors, the threshold for achieving Covid-19 herd immunity lies between 55 and 82% of the population (26).

Even though 86.7% of parents in our study are vaccinated against Covid-19, only 47.6% of the parents intend to vaccinate their children against Covid-19. This controversy was observed in various international studies. Skjefte et al. reported rates among mothers across 16 countries: above 85% in India, Mexico, Brazil, and Colombia, and below 52% for Australia, US, and Russia (9). Another study from Jordan showed that 36% of the parents agreed to receive the vaccine, but only 18% accepted it for their children (27). A study from the UK and Germany found that parents of children younger than 18 years were more hesitant to have their child vaccinated than to get vaccinated themselves (28, 29). Interestingly, in our study, parents who had received the COVID-19 vaccine themselves were found to be significantly more predicted to agree to vaccinating their children compared to those not vaccinated against COVID-19; consistent with other studies (28–31).

We intended in our survey to assess parents' intentions to vaccinate their children vs. their attitudes/beliefs toward the newly-introduced Covid-19 vaccine using the standard VHS. Intentions are defined by the person's subjective probability of doing an action while attitudes are defined by the person's favorableness or non-favorableness toward an action, or subject while beliefs are defined by person's link between two different subjects (32). Therefore, hesitancy to vaccinate children is affected directly by parents' attitudes and beliefs; however, this wouldn't reflect, necessarily, on their intentions when applied to their actions. Our results have shown significant linear correlation between both; even those who were unsure in their intention were trending more toward a positive attitude to the vaccine, as evidenced by their mean VHS.

Parents who had at least one member of their direct family who had contracted the Covid-19 disease demonstrated more intention to vaccinate their child against Covid-19, which mirrored a similar observation in another study (30). Interestingly, the severity of the family member's Covid-19 disease did not affect their decision. Parents with higher compliance to physical distancing and other precautionary measures have been described as a strong predictor of self and parental vaccine acceptance in multiple studies; Skjefte et al. reported that Covid-19 pandemic precaution measures were one important predictor for parents' acceptance of the Covid-19 vaccine (9). Zhang et al. also demonstrated lower parental acceptance of the Covid-19 vaccination in those with lower compliance to physical distancing measures and vice versa (31). However, this was not observed in our study; this may be because our surveyed population believed in the precautionary measures as a means of protection for that age group much more than the vaccine, as their compliance with them reached 90%.

We found that parental VH is more pronounced with the COVID vaccine than with routine childhood vaccines; parents' main reasons for refusal were poor safety data and concern over side effects. Similar findings were reported by Yilmaz and Sahin (30). At the same time, the parents' behaviors toward both vaccines linearly correlated in a positive manner, which was observed in previous studies; this is an expected protective behavior by caring parents, as evidenced by studies that reported that a willingness for the Covid-19 vaccination was affected by the compliance with the other vaccines in the current vaccination schedule (31, 33). A previous study showed that an expedited Covid-19 vaccine was more acceptable among those with children who completed the scheduled vaccination (34).

An interesting observation is the large increment in parents' VH rate to routine childhood vaccines (45.3 vs. 20%) as compared to what was reported in 2019 (23). While this change can be explained partly by the different methodology used to assess VH in our study compared to the previous study, other factors need to be checked. Although parents still believe that childhood routine vaccines are essential for their children's health, a majority of them believe their children do not need vaccination for diseases that are not common anymore (35).

The need to have reliable information and the dependence on sources that provide such information are of paramount importance for any health-related conditions. This is of particular importance in the case of Covid-19, where there is constant change in the information and counter-information. Misinformation, especially that specific to social media, may affect the individual's behaviors and attitudes. Our study showed that 85.9% of the parents relied on the Saudi MOH as a source for Covid-19 information. This finding is consistent with previous studies that showed 68.7% of those with Covid-19 were dependent on the Saudi MOH instructions, and that reliable sources on vaccines improved vaccine uptake (18, 36). The second most frequently relied-upon site was the WHO website. A previous study of the attitudes toward adolescent vaccination in the USA showed that having more information about the adolescent Covid-19 vaccine was associated with higher vaccine acceptance rates (37). Another study showed that exposure to negative Covid-19 vaccination information negatively impacted vaccination uptake (31).

While relying on the WHO as an information source negatively affected Covid-19 vaccination decisions in our study, the MOH as an information source had a strongly positive impact, which highlights the success of local authorities in motivating the population for vaccination. Therefore, message-tailoring directed to specific portions of societies could be a more effective way to promote vaccine acceptance. Tailored vaccine educational programs have been shown to be a more effective way to improve vaccine compliance among vaccine hesitant parents toward childhood vaccines (38, 39).

Parental age was significantly positively associated with a higher intention to vaccinate their children for Covid-19. However, their gender did not make a significant difference, which contradicts the findings of the group by Goldman et al., who surveyed about 1,000 caregivers for Covid-19 vaccine uptake of their children and found a significant tendency among fathers to vaccinate compared to mothers (34). This behavior by fathers has been described in multiple studies as being related to their personality of risk-taking behavior which was also very evident for the H1N1 vaccine when it emerged as a new epidemic (40).

In our data, education level correlated inversely with parents' intention to vaccinate their children for COVD-19; literature has been inconsistent in that regard (41, 42). This inconsistency has been previously reported when evaluating the education levels of parents and vaccine acceptance, which translate into that education cannot always be relied upon as a predictor of vaccine acceptance or more accurate knowledge on vaccines (43). Parents who received the Covid-19 vaccine shot themselves significantly had higher intention to vaccinate their children which echoes other studies that showed caregiver receiving influenza vaccine had higher intention to vaccinate their children (34).

## Study Limitations and Strengths

Our study demonstrated several strengths and limitations. It is one of the first studies to address parents' willingness/intention and hesitancy to give their children the Covid-19 vaccine, especially after the recent approval of the mRNA vaccine for the 12–18 years age group in several countries. Our large sample size with its wide spectrum of representation provided more insight for a newly emerging disease and the ongoing debate regarding its vaccine. Our survey also compared the hesitancy toward routine childhood vaccines, while addressing the psychological conflict in the minds of parents.

One of the limitations of our study was that we did not address parents' intention to vaccinate for Covid-19 for children with chronic medical illnesses or those with immune compromised conditions, as that would be a more challenging decision for parents. Also, we did not address all potential factors that contributed to parents' hesitancy for the Covid-19 vaccine, such as the differing severity of SARS-CoV-2 infections in adults as compared to children. While the Snowball sampling through social media platforms could limit its representativeness; however, this research provides a template for similar research in other countries and populations.

## Conclusion

As parental intention to vaccinate their children is suboptimal, further communication about Covid-19 vaccine safety in as the source of information was associated with increased acceptance of childhood Covid-19 vaccination; therefore, local healthcare authorities should intensify their campaigns targeting hesitant parents to increase vaccine uptake in children children is warranted. The main reasons for not intending to vaccinate their children were the inadequacy of information about the safety of the Covid-19 vaccines and worry about potential side effects. These findings call for further communication about the safety and targeting the younger parents, as they were less likely to vaccinate their children with Covid-19 vaccines. Relying on the national official healthcare authority's website.

## Data Availability Statement

Data will be made available upon reasonable request. Requests to access the datasets should be directed to mtemsah@ksu.edu.sa.

## Ethics Statement

The studies involving human participants were reviewed and approved by Institutional Review Board, King Saud University, Riyadh, Saudi Arabia. Written informed consent for participation was not required for this study in accordance with the national legislation and the institutional requirements.

## Author Contributions

M-HT: research project conceptualization and initiation, survey development, IRB approval, data collection, and manuscript drafting and finalization. ANA, FAlj, and FAB: data collection, results analysis and manuscript drafting and finalization. FB: survey development, literature review, data collection, and manuscript drafting and finalization. AAlr, AAlha, AAls, RB, FAls, AAla, and MAB: data collection and manuscript drafting and finalization. AA-E: methods and results analysis, manuscript drafting and finalization. OT, RA, YC, AJ, SA-S, JA-T, KA: research project conceptualization and initiation, survey development, introduction, method. BS, RH, FAlZ, ZM, and MB: manuscript finalization. All authors contributed to the article and approved the submitted version.

## Funding

This study was supported by King Saud University, Deanship of Scientific Research, Research Chair for Evidence-Based Health Care and Knowledge.

## Conflict of Interest

The authors declare that the research was conducted in the absence of any commercial or financial relationships that could be construed as a potential conflict of interest.

## Publisher's Note

All claims expressed in this article are solely those of the authors and do not necessarily represent those of their affiliated organizations, or those of the publisher, the editors and the reviewers. Any product that may be evaluated in this article, or claim that may be made by its manufacturer, is not guaranteed or endorsed by the publisher.

## Appendix

**TABLE A1:** Paired samples t-test comparing parental attitudes toward routine and COVID-19 children's vaccination.

**Mean (SD)- Attitude to routine children's vaccine**	**Mean (SD)- Attitude toward COVID children's vaccine Mean difference (95% C.I)**	**Mean difference (95% C.I)**	**t/df**	**p-value**
2.98 (0.58)	2.63 (0.73)	0.355 (0.338: 0.373)	40.70/3166	<0.001

*The paired samples t-test was used to compare the parental mean attitudes toward routine and COVID19 vaccines as measured with the personalized VHS questionnaire. The resulting findings in Table 2 showed that the parents had significantly greater positive attitudes toward the children's routine vaccines (mean attitude = 2.98/4) compared to their attitudes toward the children's COVID19 vaccines (mean attitude = 2.63) on average, denoting that they had stronger agreement with the children's routine vaccines than the COVID19 vaccines in general, mean difference = 0.355 (95% C.I mean difference: 0.338-0.377), t = 40.70, df = 3, 166, p-value < 0.001*.

## Abbreviations

CDC: Centers for Disease Control and Prevention
Covid-19: Coronavirus disease 2019
GAD-7: Generalized Anxiety Disorder Assessment
MOH: Ministry of Health
SARS-CoV-2: Severe Acute Respiratory Syndrome Coronavirus 2
VHS: Vaccine Hesitancy Scale
WHO: World Health Organization.
